# Analysis of the Impact of Environmental Perception on the Health Status of Middle-Aged and Older Adults: A Study Based on CFPS 2020 Data

**DOI:** 10.3390/ijerph20032422

**Published:** 2023-01-30

**Authors:** Tingyi Liu, Huake Liu, Shibing You

**Affiliations:** School of Economics and Management, Wuhan University, Wuhan 430072, China

**Keywords:** environmental perception, depression, middle-aged and older adults, health status, logistic regression, PSM

## Abstract

Health risks and hazards caused by the environment have long been one of the most important public issues of concern to the state, society, and the public. At the same time, population aging is becoming a global issue, and residents’ health is the most important component of people’s livelihood, and residents can only pursue other rights and interests if they can protect their own health. Therefore, based on the micro data from the fifth round of the China Family Panel Studies (CFPS), this paper uses binary logistic regression with propensity score matching (PSM) to analyze the effect of environmental perception on the health status (including mental health and physical health) of middle-aged and older adults. It was found that environmental perceptions significantly affect the depressive state and sickness status of middle-aged and older adults. Among them, middle-aged and older adults who were female, of rural households, with low education and relatively low income were more affected by environmental shocks on their health. Therefore, we should pay attention to the mental and physical health of middle-aged and older adults and change the existing design concept of aging policy: the government should formulate effective policies and increase corresponding social support; and society and families should also give corresponding care and encourage middle-aged and older adults to exercise more and provide reasonable psychological guidance.

## 1. Introduction

### 1.1. Background of the Study

In recent years, China’s development has been characterized by continuous and rapid development on the one hand, and on the other hand, by challenges of an environmental nature.

Sustainability is among the most complex and difficult issues in the world. Health risks and hazards caused by the environment have also long been one of the public topics of greatest concern to the state, society and the public. At the same time, population aging is becoming a global issue, and residents’ health is the most important component of people’s livelihood, and residents can only pursue other rights and interests if they can protect their own health. In China, middle-aged and older adults are more likely to suffer from health problems due to their low socioeconomic status and physical deterioration. Compared with young people, middle-aged and older adults’ health levels are more influenced by their nearby environment due to their limited mobility; therefore, the health problems of middle-aged and older adults have attracted more attention [1]. Developing countries, in particular, are not yet adequately prepared to deal with these problems [2]. The Chinese government and leaders have emphasized in several documents and speeches that “environmental protection and governance should focus on solving environmental problems that are detrimental to the health of the public”. However, the severe resource and environmental situation not only raises serious public concerns about environmental health but also further exacerbates social problems such as the gap between the rich and the poor and social injustice. When environmental pollution and health, income and other issues are intertwined and affect each other, it is very likely to fall into the “environmental health poverty trap”: environmental pollution damage to health–induce disease–damage to labor capacity–aggravate the economic trap [3,4]. Therefore, it is important for national life to explore whether environmental perceptions have an impact on the health of middle-aged and older adults. If so, are there differences in the effects of environmental perceptions on the health of different groups of residents? To obtain relevant conclusions, this paper develops an analysis of the effect of environmental perception on the health status of middle-aged and older adults.

### 1.2. Purpose and Significance of the Study

Health is an inevitable requirement for promoting comprehensive human development and a basic condition for economic and social development. Environmental and health issues involve multidisciplinary interdisciplinary fields, and their identification and assessment are very complex and scientific in nature. At the same time, environment and health is also a highly concerned and sensitive social issue, which directly affects public health, national quality, and survival and reproduction, and is even related to social harmony and stability and national security. Therefore, the purpose of this paper is to study the degree of influence of environmental perceptions on residents’ health from a multidisciplinary perspective, to investigate the effects of each influencing factor and to provide a relevant basis for the overall improvement of health.

Since the birth of human civilization, health has been the goal of human beings, and people are more and more eager to have a healthy living environment. As “Healthy China” has become a national development strategy, the main task of environment and health work is to reveal the occurrence and development pattern of environmental factors on the health of people. Therefore, studying the differences of environmental perception on the health of different groups through microscopic data can fully understand the reality and provide some ideas or perspectives for macroscopic decision-making.

## 2. Literature Review

Health is a changing process with dynamic characteristics, and its concept is influenced by the productivity, production relations, technology level, and philosophical thought at a certain historical stage. It is constantly updated with the change of times, and its connotation is enriched day by day. The evaluation of individuals by health status is the result of the joint action of natural and social environment. According to the relevant literature, the health status of middle-aged and older adults can be reflected from two aspects: subjective (psychological) and objective (physical). The objective aspect of health status, reflected from the physiological perspective, focuses on studies of environmental effects on health status, mostly focusing on certain serious diseases as important phenotypes, for example, hypertension, cancer, pneumoconiosis, etc. The subjective aspect of health status is represented from the psychological perspective, and the prevalence of depression as a typical psychiatric disorder is at its peak among middle-aged and older adults, and depression among the health problems of middle-aged and older adults has attracted widespread research attention due to its increasing rate, a major chronic disease that imposes a heavy burden on many countries and families [5]. In addition to causing the patients’ own mental suffering, depressive mood can trigger a host of other health problems, such as type II diabetes and cardiovascular disease, which further lead to secondary comorbidities and make many families face great challenges financially and mentally. Factors such as [6] illiteracy [7], marital status [8], unhealthy lifestyle habits [9], surrounding environment [10,11], built environment [12], subject behavior, and [13] neighborhood [14,15] are capable of significantly influencing health status.

Groups living in the same environment have different effects on residents of different social backgrounds and different age groups, so that factors that significantly affect residents’ health in other countries may not necessarily have a better effect in China. Based on this situation, it is necessary to introduce the factors mentioned by domestic and foreign scholars in order to discover the degree of influence of environmental perception on the health of middle-aged and older adults in China.

By combing through the relevant literature, the research characteristics of this paper are as follows:

(1) Although some researchers have focused on the effect of environment on depressed mood, the studies have been limited to individual cities, with less attention to other regions or even the whole country, and most researchers lack data that contain a large number of environmental perceptions with a large enough sample size and a wide enough range, thus limiting them to a particular region [16,17]. (2) The study population is adults, and there are no studies for middle-aged and older adults, so the population of this paper is the entire middle-aged and older adults’ sample who received the CFPS survey. This study will use CFPS data containing environmental perceptions, and this data covers the whole country, which provides a solid data base for this study. (3) Current studies have mainly included objectively measured environmental variables, while subjective perceived urban environmental variables can reflect health effects that are difficult to be captured by objectively measured urban environmental variables, and their specific roles need to be explored in depth. In view of this, based on sociological and economic perspectives, this paper incorporates environmental perceptions and residents’ health into a unified framework based on the CFPS database and empirical methods such as logistic regression and PSM to analyze the effect of environmental perceptions on residents’ health and proposes corresponding countermeasures based on the findings of the empirical analysis.

## 3. Data Sources and Variable Descriptions

### 3.1. Data Sources

The data used in this paper come from the China Family Panel Studies (CFPS), which is implemented by the China Social Science Survey Center of Peking University. CFPS has been officially implemented in 25 provinces/municipalities/autonomous regions in China since 2010 as a baseline survey, and 14,960 households and 42,590 individuals were completed interviews, which are conducted every two years. The questionnaire is a nationwide, comprehensive social tracking survey project that aims to reflect the social, economic, demographic, educational, and health changes in China by tracking and collecting data at the individual, household, and community levels, and providing a data base for academic and policy research.

In this paper, we use the fifth CFPS cross-sectional data in 2020 to empirically analyze the impact of individual subjective environmental perceptions on the health of middle-aged and older adults in China. The main reason for choosing middle-aged and older adults as the study population in this paper is to consider that minors under the age of 18 have weaker subjective judgment ability while young adults are relatively less influenced by the environment, whereas for middle-aged and older adults who have been exposed to the same environment for a long time, environmental perceptions play an important role in their health status. In this paper, middle-aged and older adults are defined as Chinese citizens who are at least 45 years old and have settled in China for a long time.

In addition, this paper aims to capture the dynamic relationship between environmental perception and human health through panel data, so invalid samples from the adult pool with 2020 and access not completed were excluded, and sample data with missing key variables were interpolated and selectively removed, and finally 9112 valid samples were obtained.

### 3.2. Variable Selection and Description

The health status of middle-aged and older adults can be reflected from both subjective (psychological) and objective (physical) aspects. In this paper, the objective health status of middle-aged and older adults is reflected by the question: “In the past 12 months, who took care of you most when you were not feeling well or when you were sick?”. The objective health condition of middle-aged and older adults is reflected in the study, and if someone takes care of you, it means “sick” and is recorded as “1”, while the rest of the options are recorded as “0”. The environment is closely related to people’s lives, and a poor living environment makes people prone to anxiety and depression, and studies have shown that depression peaks among middle-aged and older adults [18]. Objective health is mainly expressed by depressive states, where depressive states are a combination of illnesses characterized by a depressed state of mind and accompanied by emotional manifestations such as reduced pleasantness, depressed mood, loss of mood, and somatic behavioral symptoms. Depression was measured in the questionnaire using the CES-D, and the respondents’ mental health status was reflected by investigating eight questions. Firstly, the positive indicators of the CES-D were reverse coded; secondly, the depression scale options in the database were “1. hardly ever (less than one day), 2. some of the time (1–2 days), 3. often (3–4 days), 4. most of the time (5–7 days)” with a full score of 32. According to the international criterion, 0–16 is not depressed and 17–32 is depressed [19].

The explanatory variable of this paper is the respondents’ evaluation of the environment, which is determined by the questionnaire: “How serious do you think the environmental problems are in our country”. The higher the respondents’ scores in this variable, the higher the degree of dissatisfaction with the current state of the environment, and this paper focuses on the effect of environmental perception shock on the health status of middle-aged and older adults. The control variables were selected from demographic and daily life perspectives, including gender, age, urban and rural distribution, education level, marital status, life satisfaction, smoking, drinking, and exercise. The detailed variable treatments and variable naming are shown in Table 1.

### 3.3. Model Construction

In this paper, the variables reflecting the health status of middle-aged and older adults are treated as dichotomous variables, and binary logistic regression is used to explore the effect of environmental perception on the health of middle-aged and older adults.

In the binary logistic model under the influence of factor *X*, the probability of event occurrence can be expressed as:

(1) $$P(Y=1/X)=\frac{e^{\left(\beta_{0}+\beta_{1}\times x_{1}+\beta_{2}\times x_{2}+\ldots +\beta_{n}\times x_{n}\right)}}{1+e^{\left(\beta_{0}+\beta_{1}\times x_{1}+\beta_{2}\times x_{2}+\ldots +\beta_{n}\times x_{n}\right)}}$$

where *Y* is represented by “0” and “1”, which represent the two outcomes of the event, and the constant term, which is the regression coefficient under each influencing factor.

After Logit transformation, we get:

(2) $$\ln(\frac{P(Y=1/X)}{1-P(Y=1/X)})=\beta_{0}+\beta_{1}\times x_{1}+\beta_{2}\times x_{2}+\ldots +\beta_{n}\times x_{n}$$

The propensity score method combines multiple covariates into a single indicator variable, which is called the propensity score and is used to represent the joint effect of multiple covariates. When the propensity scores of two study groups are the same, it indicates that the covariates show a balanced distribution between the two groups. The expression for the propensity score is:

(3) $$E(x_{i})=P(Z_{i}=1|x_{i})$$

This expression represents the conditional probability that the ith object is assigned to the treatment group when a set of covariates is known to exist. Where *x_i_* denotes the known covariates of the *i*(*i* = 1, 2, …, *N*) object under study, indicating that the object is assigned to the treatment group. The above model practical operations are performed by STATA.

## 4. Analysis of the Impact of Environmental Perception on the Health Status of Middle-Aged and Older Adults

### 4.1. Descriptive Statistical Analysis

In this study, there are 9112 total samples, among which 4492 people are dissatisfied with the current environmental situation and think that the current environmental problems in China are more serious, accounting for 49.3% of the total sample, which is almost half of the total sample and reflects the seriousness of the environmental problems; among the respondents, there are 4884 men, accounting for 53.6% of the total sample. The sample size is reasonable and the data structure is appropriate. The descriptive statistics of the relevant variables are shown in Table 2, where: column (1) is the descriptive statistics of the total sample as a whole; columns (2) and (3) are the descriptive statistics classified by treatment group; and columns (4) and (5) are the descriptive statistics classified by gender. The average age of respondents overall is about 57 years old, and the average age of those who think environmental problems are more serious is 56 years old; the average age of female respondents is 56 years old, which is about 0.9 years lower than the average age of male respondents, and their depression score is about 1.2 points higher than that of men, which shows that women are more likely to fall into producing depression compared to men.

### 4.2. Binary Logistic Regression Analysis

A binary logistic regression analysis was conducted using the input method to analyze the factors affecting the health of middle-aged and older adults. Relevant tests are required before obtaining the regression results. The comprehensive tests of the model coefficients are shown in Table 3, and the *p*-values are less than the significance level of 0.05, indicating that the model is statistically significant in general. The results of the Hosmer and Lemeshow tests are shown in Table 4, and the *p*-values are greater than the significance level, rejecting the original hypothesis, indicating that there is no difference between the predicted and true values, and that the prediction of the established model fits well. In summary, according to the test results of the regression model, it is clear that the model established by using the binary logistic regression method is better.

The results of the logistic regression analysis are shown in Table 5, and the model was constructed according to the coefficient table as follows:

(4) $$\begin{array}{l} Logit(P_{dep})=0.124env-0.385gender-0.027age-0.253urban\\ -0.311edu-0.204word-0.705marr-0.419income+0.142smoke\\ -0.263drink-0.270sport+0.350 \end{array}$$

(5) $$\begin{array}{l} Logit(P_{sick})=0.183env-0.266gender+0.420age-0.172urban\\ -0.208edu-0.212word-0.082marr-0.190income-0.172smoke\\ -0.300drink+0.123sport+2.026 \end{array}$$

In terms of subjective and objective health, the model results showed that the sig value of the effect of age on mental health was 0.623, and the sig values of marital status and exercise frequency on physical health were 0.446 and 0.071, respectively, which were greater than the significance level of 0.05, which means that the effect of age change on the mental health status of middle-aged and older adults, and the effect of marital status and exercise frequency on physical health was considered insignificant. Among them, in terms of subjective health, the effects of gender, urban and rural distribution, education, literacy, marital status, income satisfaction, alcohol consumption, and exercise on mental health were negative, and positive relationships existed between perception of environment and smoking on mental health.

In terms of objective health, perception of environment, age, and exercise had significant positive effects on the health status of middle-aged and older adults, and effective and moderate exercise could reduce the frequency of illness among middle-aged and older adults. The influence of environmental perception indicators on both subjective and objective health status of the survey respondents was significant and both showed a positive influence relationship, and people with poorer evaluation of environmental conditions had poorer subjective and objective health status.

From the analysis results in the table, it can be seen that the probability of risk of depression for people with poor evaluation of environment is 1.132 times that of people with excellent environmental evaluation, and the probability of possible disease is 1.201 times higher, that is, the higher the perceived degree of environmental pollution, the worse the health condition of people. Therefore, relevant authorities should pay attention to environmental governance and propose policies to enhance environmental regulation.

Women are at higher risk of experiencing both mental health and physical health hazards than men in the Health Rating Scale. Specifically, all else being equal, women are 31.9% more likely than men to suffer from depression in middle-aged and older adults, while women are 23.4% more likely than men to suffer from disease due to their relatively poor physical health. Men are generally perceived to be more physically fit than women, and men play a major productive role in society.

Compared to the rural group, the urban group is 22.4% less likely to suffer from depression and 15.8% less likely to suffer from disease. The living environment of the middle-aged and older adults in the urban group is to some extent better than that of the rural group, which is weaker in terms of medical and sports infrastructures.

At the same time, groups with high education, literacy, and satisfaction with job income have lower probability of suffering from both mental and physical diseases, and the higher the education level, the greater their awareness and concern for health and the higher the physical health return rate, which can actively improve their health and is consistent with the reality.

The results of the model analysis show that the degree of concern for physical health of middle-aged and older adults in different age groups is different. With the increase of age, people pay more and more attention to health, and the physical health of a person will gradually decline in the opposite direction. Relative to middle-aged people, the older they are, the greater the probability of disease, and the probability of disease is 1.521 times of the reference group of middle-aged people, which is in line with the natural law. The mental health status of the group with spouses was better than that of the group without spouses, indicating that the marital status of the elderly is one of the important factors that cannot be ignored in their health status and that the presence of a spouse in later life is a boost to the life and health of the elderly.

Smoking, drinking, and exercise have a certain degree of influence on the health status of middle-aged and older adults. The probability of getting depression is 1.153 times higher in the middle-aged and older adults who smoke than those who do not smoke, and drinking and exercise can reduce the probability of getting depression by 23.1% and 23.6%. Physical exercise has a good fitness effect, and regular sports exchange and demonstration activities can stimulate middle-aged and older adults’ enthusiasm to participate in physical exercise, while the cost is low, which can improve the psychological health of the elderly.

### 4.3. Propensity Score Matching PSM Analysis

Considering the endogeneity problem caused by sample selection bias and omission of variables in estimating the effect of environmental conditions on the health status of middle-aged and older adults, propensity score matching analysis was further performed on the sample, where the group with worse environmental conditions was considered as the treatment group. Two important assumptions need to be met before matching the CFPS2020 data: (1) given the covariate X, the treatment group’s depression and illness status and the control group’s depression and illness status are independent of the environmental shock, which is randomized; (2) there is an overlap between the treatment and control group samples to ensure that the two groups have the same range of propensity score values of the two groups.

A two-step approach was used for propensity score matching. In the first step, the control variables mentioned above were used as observable covariates, namely gender, age, urban, education, word, marriage, income, smoke, drink, and sport ten variables, all of which would have a corresponding effect on the health status of middle-aged and older adults. The selection of this covariate can balance the treatment group and the control group, so that the two groups are as similar as possible in these ten characteristics, which can eliminate the self-selection problem of the sample to some extent. Logit regression with 0.02 caliper was set for propensity score matching, and we performed one-to-one nearest neighbor matching on the samples, and a total of 4069 middle-aged and older adults were matched to the control samples among 4492 samples of the treatment group, which contained 8 pairs of precise matching and 4061 pairs of fuzzy matching, and only 423 samples were not matched to the data, and the sample matching rate was high, and the matching results are shown in Table 6.

**Figure 1:**
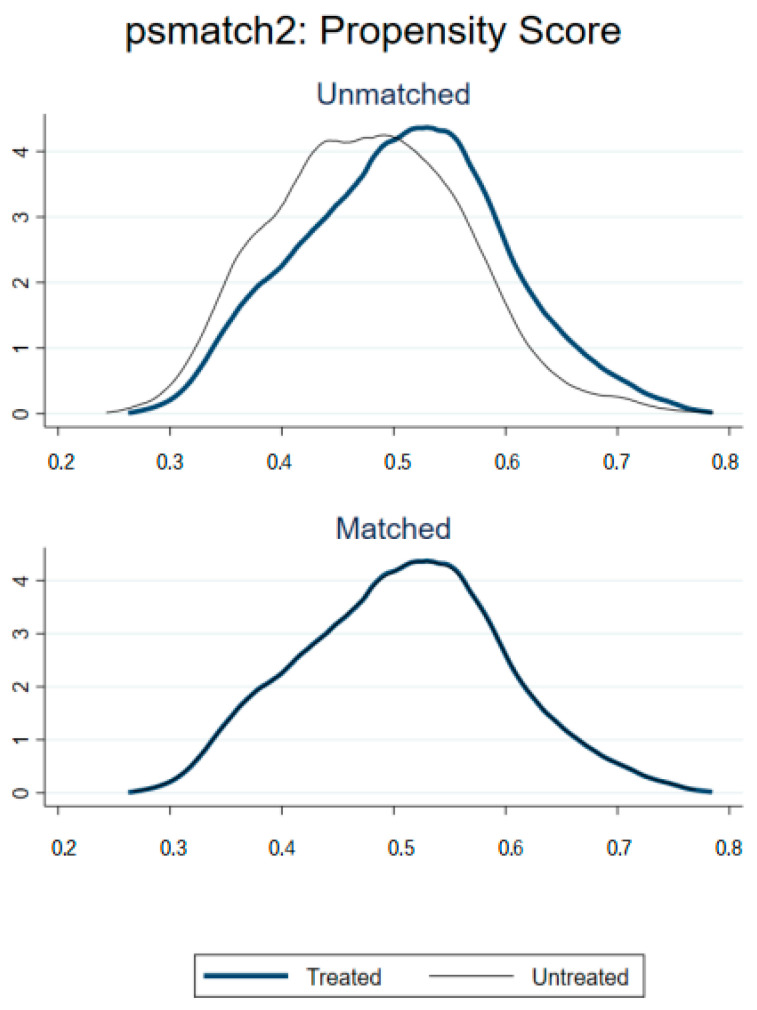
The kdensity density distribution before and after sample matching.

**Figure 2:**
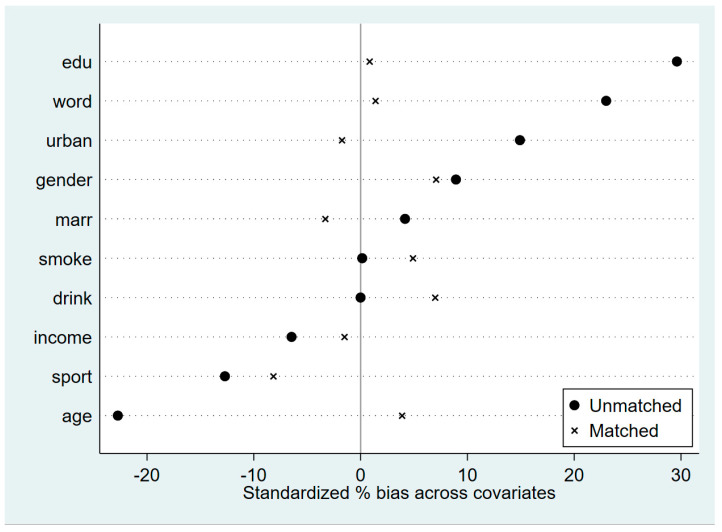
Standardized deviation before and after covariate matching.

The nearest neighbor matching results of the samples are shown in Table 7 below, and the kdensity density distribution of the samples before and after matching is shown in Figure 1. As can be seen from the results in the figure, the difference between the treatment and control groups before matching is large, while the difference between the treatment and control groups becomes significantly smaller after matching. Figure 2 shows the standardized deviations of the covariates before and after matching, and it is found that the standardized deviations of each covariate after matching are smaller than those before matching, and the standardized deviation values of all covariates do not exceed 10%. This shows that there is no significant difference between the observable characteristics of the treatment and control groups after matching, and the control results are convincing.

After propensity score matching, the results were analyzed as follows: Table 8 results showed that, consistent with the binary logistic regression results, the t-values of the ATT results for the status of being depressed or not and the status of being sick were both greater than 1.96, indicating that environmental perceptions had a significant effect on both mental health and physical health of middle-aged and elderly people at the 0.05 level.

### 4.4. Heterogeneity Analysis

The effects of environmental pollution on population health may vary by group type. In this paper, group regressions were conducted for different age and gender groups to investigate whether there are differences in the transmission of environmental perceptions among different groups through heterogeneity analysis. The results from the above analysis indicated that the perceived level of environmental pollution status negatively affected the health status of middle-aged and older adults, but after controlling for individual-related heterogeneity factors, environmental perceptions were found to have a significant effect on the physical and mental health status of middle-aged and older adults of different age and gender groups.

Table 9 and Table 10 confirm the existence of heterogeneity from both age and gender perspectives, respectively. The analysis of the regression coefficients shows that the middle-aged group aged 45–59 is relatively more resistant to the same environmental pollution compared to the older group aged 60 and above. Although environmental pollution has a negative impact on both the mental health and physical health of middle-aged and older adults, and the results are significant at the 10% and 5% levels, respectively, the impact is significantly smaller for the younger age group. It indicates that the health condition of the elderly group is more affected by the environment and focusing on the protection of the environment should become a topic of social concern.

The effects of environmental pollution on population health may vary by gender due to differences in body structure and lifestyle, and this paper tests the heterogeneity of environmental pollution on the physical and mental health of men and women. The results show that the perception of environmental pollution has a significant negative impact on the mental health of the middle-aged and older adults groups, and the impact on the female group is relatively greater. From a physical health perspective, among the middle-aged and elderly population, men are more affected by air pollution compared to women, probably due to the relatively longer time spent outdoors by men.

### 4.5. Robustness Test

Two other measures of the health status of middle-aged and older adults exist in the questionnaire: “How much do you rate your confidence level in the future?” and “How do you think your health condition is?” Therefore, the core variables were replaced in this paper, and robustness tests were conducted, and the results are shown in Table 11. The test model shows that environmental pollution status has a significant positive effect on both self-rated health and future confidence of middle-aged and older adults at the 10% level, and the conclusion of the model before and after replacing the core variables is consistent with the previous regression model that environmental pollution can negatively affect physical and mental health, so the government should formulate relevant public health policies to reduce the impact of environmental pollution on residents’ health.

## 5. Conclusions and Recommendations

After analysis, it can be seen that the explanatory and control variables selected in this paper have significant effects on the overall health status of middle-aged and older adults in China, and each influencing factor has direct or indirect effects on each other and shows an intricate relationship with each other. The degree of environmental perception of the survey respondents had a significant and important impact on psychological and physical health, and with age, the probability of risk of depression was 1.132 times higher for those with poor environmental evaluation than for those with excellent environmental evaluation, and the probability of possible illness was 1.201 times higher. With increasing age, various organs of the human body change, chronic diseases emerge in middle and old age, and physiological health is poor. Among them, the overall health status of urban residents is better than that of rural groups, while the mental and physical health status of men is better than that of women. Education can improve middle-aged and older adults’ perception of their health level, while the higher satisfaction with their income and the presence of a spouse, the higher the degree of economy and the more they can pursue a healthy and productive lifestyle. Smoking, drinking, and exercise also have an impact on the health status of middle-aged and older adults.

After propensity score matching, the impact of environmental status was analyzed under the condition of matching covariates, which further verified the valid conclusion obtained from the binary logistic regression that environmental status in China has a significant impact on both mental health and physical health of middle-aged and older adults.

The reliability of the conclusion that increased environmental pollution will pose a threat to the physical health and mental health of middle-aged and older adults was further verified by heterogeneity analysis and robustness type test. Meanwhile, environmental pollution had a significant positive effect on the physical and mental health of elderly people of different genders and ages, but there was a slight difference in the degree of effect, with a greater effect on the older elderly than the younger elderly. The psychological health of women was affected to a greater extent than that of men, and the physical health of women was affected to a lesser extent than that of men.

In response to the conclusions obtained from the analysis, the following recommendations are made. Since the higher the perception of environmental pollution, the worse the health condition of people, therefore, with the development of society, environmental pollution is very harmful to human life as well as the development of society, and human diseases caused by environmental pollution factors such as air, water, soil, and electronic radiation are very common. The government and relevant departments should strengthen their leadership and guidance, establish a sound legal system and policy orientation for ecological and environmental green development, and gradually build an economic system of environmental protection and low carbon cycle sustainable development. At the same time, they should also pay attention to the improvement of agricultural and industrial production processes, establish a comprehensive monitoring system for environment and health, strengthen environmental and health investigation and research and information platform construction, accelerate the establishment of environmental and health risk management mechanisms as well as enhance the level of public environmental and health literacy, reduce the emission of pollutants, and continuously build an ecological civilized society.

The household registration, age, gender, marital status, and income satisfaction of middle-aged and elderly people are established facts, and it is unrealistic to change them in the short term through human factors. However, it is possible to improve the “education” of middle-aged and older adults, and local governments and public welfare institutions can establish senior citizen universities to support them to continue their studies. The rich and diversified courses offered by the universities can help to relieve their depression and improve their health satisfaction.

Middle-aged and older adults should be actively encouraged to persist in exercise and actively guided to participate in social activities. Regular physical exercise can improve the immunity and energy of middle-aged and older adults, which is good for relieving illnesses, improving sleep, and enhancing physical strength. The personal behavior of middle-aged and older adults is also important. Communities can mobilize the subjective initiative of middle-aged and elderly people to participate in social activities by setting up dance teams, chess and card clubs, and organizing activities regularly to promote middle-aged and older adults to face life positively.

Urban residents have better access to medical and health care than rural groups, so the infrastructure construction of rural medical and health service programs should be increased, and middle-aged and older adults can experience perfect and advanced facility services to enrich their needs. Meanwhile, the community should hold more health lectures about middle-aged and older adults, regularly carry out group recreational activities, and the staff of the neighborhood committee should regularly visit households to record and monitor the health status of middle-aged and older adults and provide targeted psychological guidance to middle-aged and older adults with poor health status and low health satisfaction.

## Figures and Tables

**Table 1:** Variable Design.

Variable Category	Variable Name	Variable Correspondence Problem	Variable-Assisted Processing
Explained variables	cesd8	I feel emotionally drained. I find it a struggle to do anything. I don’t sleep well. I feel pleasant. I feel lonely. I live a happy life. I feel sad and miserable. I feel like life can’t go on.	Score
	dep	Whether depressed or not	No depression = 0 Depression = 1
	sick	In the past 12 months, who was your primary caregiver when you were not feeling well or when you were sick? If there were multiple caregivers, please select the primary one.	No sickness = 0 Others = 1
Explanatory variables	env	How serious do you think the environmental problems are in our country (0 means not serious, 10 means very serious)	0–5 = 0 6–10 = 1
Control variables	gender	Respondents’ gender	Female = 0 Male = 1
	age	Respondents’ age	45–59 years = 0 Other = 1
	urban	Urban–rural classification based on information from the National Bureau of Statistics	Rural = 0 Other = 1
	education	Highest education in the last survey	Illiterate/semi-literate, did not go to school, elementary school = 0 Others = 1
	word	Literate or not	Illiterate/semi-literate = 0 Others = 1
	marriage	Latest survey on marital status	No spouse (unmarried, divorced, widowed) = 0 Others = 1
	income	How satisfied are you with your income from this job?	Dissatisfied (very dissatisfied, not very satisfied, average) = 0 Others = 1
	smoke	Have you smoked in the past month?	No = 0 Yes = 1
	drink	Do you drink alcohol more than 3 times a week?	No = 0 Yes = 1
	sport	How often did you participate in sports, fitness, and leisure activities in the past 12 months?	Never attended, average less than 1 time per month, average more than 1 time per month but less than 1 time per week, average 1–2 times per week = 0 Others = 1

**Table 2:** Variable Descriptive Statistics.

	(1)	(2)	(3)	(4)	(5)
Variable	Overall Sample	Treated	Controls	Male	Female
gender	0.536	0.559	0.514	1.000	0.000
age	57.104	56.141	58.040	57.537	56.603
urban	0.443	0.480	0.406	0.442	0.444
edu	3.114	3.346	2.888	3.439	2.737
word	0.713	0.766	0.662	0.811	0.601
marr	0.922	0.927	0.916	0.931	0.911
income	3.467	3.430	3.502	3.502	3.426
smoke	0.314	0.314	0.313	0.560	0.029
drink	0.171	0.171	0.171	0.291	0.032
sport	7.079	6.973	7.183	7.064	7.097
env	0.493	1.000	0.000	0.514	0.469
cesd8	13.783	13.768	13.797	13.224	14.429
dep	0.250	0.252	0.248	0.208	0.298
hea	0.667	0.690	0.645	0.711	0.616
sick	0.813	0.819	0.807	0.779	0.852

**Table 3:** Comprehensive Test of Model Coefficients.

Model	Chi-Square Value	df	Sig
dep	414.597	11	0.000
sick	246.340	11	0.000

**Table 4:** Hosmer and Lemeshow Test.

Model	Chi-Square Value	df	Sig
dep	12.853	8	0.117
sick	8.487	8	0.387

**Table 5:** Analysis of factors affecting the health of middle-aged and older adults.

Variables	Dep				Sick			
	B	Wald	Sig	Exp(B)	B	Wald	Sig	Exp(B)
env	0.124	6.095	0.014	1.132	0.183	10.962	0.001	1.201
gender	−0.385	34.239	0.000	0.681	−0.266	13.821	0.000	0.766
age	−0.027	0.241	0.623	0.974	0.420	44.546	0.000	1.521
urban	−0.253	23.178	0.000	0.776	−0.172	9.068	0.003	0.842
education	−0.311	24.101	0.000	0.733	−0.218	10.312	0.001	0.804
word	−0.204	9.522	0.002	0.816	−0.212	6.731	0.009	0.809
marriage	−0.705	72.031	0.000	0.494	−0.082	0.581	0.446	0.921
income	−0.419	70.198	0.000	0.658	−0.190	11.394	0.001	0.827
smoke	0.142	4.268	0.039	1.153	−0.172	6.258	0.012	0.842
drink	−0.263	11.969	0.001	0.769	−0.300	17.871	0.000	0.741
sport	−0.270	17.253	0.000	0.764	0.123	3.263	0.071	1.131
Constants	0.350	12.172	0.000	1.419	2.026	250.519	0.000	7.582

**Table 6:** Case Control Match Statistics.

Match Type	Count
exact match	8
fuzzy matching	4061
mismatch (including missing keys)	423
does not match (key is valid)	423
sampling	no replacement function
log file	none
Maximize matching performance	yes

**Table 7:** Nearest neighbor matching results.

env	Coef.	Std. Err.	z	*p* > z
gender	0.198	0.055	3.570	0.000
age	−0.020	0.003	−7.450	0.000
urban	0.125	0.045	2.770	0.006
edu	0.191	0.028	6.700	0.000
word	−0.218	0.094	−2.320	0.021
marr	0.021	0.080	0.260	0.796
income	−0.042	0.019	−2.160	0.031
smoke	−0.121	0.056	−2.140	0.032
drink	−0.065	0.060	−1.070	0.285
sport	−0.026	0.014	−1.890	0.059
_cons	0.871	0.223	3.910	0.000
Log likelihood	−6169.9701		Pseudo R^2^	0.023

**Table 8:** Propensity Score Match Estimation Results.

Variable	Sample	Treated	Controls	Difference	S.E.	T-Stat
dep	Unmatched	0.252	0.248	0.003	0.009	0.340
	ATT	0.252	0.225	0.027	0.012	2.200
	ATU	0.248	0.271	0.023		
	ATE	0.025				
sick	Unmatched	0.819	0.807	0.011	0.008	1.400
	ATT	0.819	0.791	0.028	0.011	2.550
	ATU	0.807	0.840	0.033		
	ATE	0.030				

**Table 9:** The impact of environmental perception on the health status of different age groups.

Variable Name	Age = 0		Age = 1	
	Dep	Sick	Dep	Sick
env	0.109 *	0.137 **	0.156 *	0.303 ***
	(0.080)	(0.036)	(0.066)	(0.004)
constants	0.401	2.055	0.325	2.400

Note: Control variables are controlled in each model. * represents significant at the 10% level, ** represents significant at the 5% level, and *** represents significant at the 1% level.

**Table 10:** The impact of environmental perception on the health status of different gender groups.

Variable Name	Gen = 0		Gen = 1	
	Dep	Sick	Dep	Sick
env	0.132 *	0.147 *	0.122 *	0.207 ***
	(0.054)	(0.097)	(0.099)	(0.003)
constants	−0.021	2.107	0.369	1.706

Note: Control variables are controlled in each model. * represents significant at the 10% level, and *** represents significant at the 1% level.

**Table 11:** Sample robustness test results.

	Replaced Core Variables	
Variable Name	How Do You Consider Yourself to Be in Good Health?	What Is Your Level of Confidence in Your Future?
env	0.091 *	0.094 *
	(0.051)	(0.061)
constants	−0.341	0.030

Note: Control variables are controlled in each model. * represents significant at the 10% level.

## Data Availability

Publicly available datasets were analyzed in this study. This data can be found here: http://www.isss.pku.edu.cn/cfps/ (accessed on 13 June 2022).
